# Seasonally Related Disruption of Metabolism by Environmental Contaminants in Male Goldfish (*Carassius auratus*)

**DOI:** 10.3389/ftox.2021.750870

**Published:** 2021-09-29

**Authors:** Lisa N. Bottalico, Julia Korlyakova, Aalim M. Weljie, Hamid R Habibi

**Affiliations:** ^1^ Department of Systems Pharmacology and Translational Therapeutics, Center of Excellence in Environmental Toxicology, Perelman School of Medicine, University of Pennsylvania, Philadelphia, PA, United States; ^2^ Department of Biological Sciences, University of Calgary, Calgary, AB, Canada

**Keywords:** metabolic and endocrine disruption, H1-NMR metabolomics, brain, liver, testis, contaminant mixture

## Abstract

Endocrine disrupting chemicals mimic or disrupt action of the natural hormones, adversely impacting hormonal function as well as cardiovascular, reproductive, and metabolic health. Goldfish are seasonal breeders with an annual reproductive cycle regulated by neuroendocrine signaling which involves allocation of metabolic energy to sustain growth and reproduction. We hypothesize that seasonal changes in physiology alter overall vulnerability of goldfish to metabolic perturbation induced by environmental contaminants. In this study, we assess effects of endogenous hormones, individual contaminants and their mixture on metabolism of goldfish at different reproductive stages. Exposure effects were assessed using ^1^H-NMR metabolomics profiling of male goldfish midbrain, gonad and liver harvested during early recrudescence (October), mid-recrudescence (February) and late recrudescence (June). Compounds assessed include bisphenol A, nonylphenol, bis(2-ethylhexyl) phthalate, fucosterol and a tertiary mixture (DEHP + NP + FS). Metabolome-level responses induced by contaminant exposure across tissues and seasons were benchmarked against responses induced by 17β-estradiol, testosterone and thyroid hormone (T3). We observe a clear seasonal dependence to metabolome-level alteration induced by hormone or contaminant exposures, with February (mid-recrudescence) the stage at which male goldfish are most vulnerable to metabolic perturbation. Responses induced by contaminant exposures differed from those induced by the natural hormones in a season-specific manner. Exposure to the tertiary mixture induced a functional gain at the level of biochemical pathways modeling over responses induced by individual components in select tissues and seasons. We demonstrate the importance of seasonally driven changes in physiology altering overall vulnerability of goldfish to metabolic perturbation induced by environmental contaminants, the relevance of which likely extends to other seasonally-breeding species.

## Introduction

Endocrine disrupting chemicals (EDCs) are chemical pollutants that mimic or disrupt action of the natural hormones, adversely impacting hormonal function as well as cardiovascular, reproductive, and metabolic health (Gore et al., 2015). EDCs signal through a variety of hormonal and nutrient sensing receptor pathways (Gore, 2010; Kassotis and Stapleton, 2019). Exposure to EDCs has been shown to disrupt a variety of physiological functions and induce obesogenic and diabetogenic effects, though specific molecular mechanisms driving metabolic perturbations remain to be elucidated (Maradonna and Carnevali, 2018; Santangeli et al., 2018; Kassotis and Stapleton, 2019).

Goldfish have been used by a number of investigators to study seasonal hormonal control of reproduction and signaling (Popesku et al., 2008; Chang et al., 2009; Ma et al., 2020a, Ma et al., 2020b) Goldfish are seasonal breeders with an annual reproductive cycle. In fall, goldfish gonad starts to grow and develop (early recrudescence), reaching the mid-stage of its development in winter (mid-recrudescence), the late stage in spring (late-recrudescence) and spawn in summer. This reproductive cycle is controlled by environmental cues affecting neuroendocrine signaling and involves a transition to allocate metabolic/energetic resources from growth to reproductive stages (Wade et al., 1996; Fernandez-Fernandez et al., 2006; Zhang et al., 2009; Ma et al., 2020a, Ma et al., 2020b; Ladisa et al., 2021).

Endogenous hormone levels and hormone receptor expression in goldfish exhibit seasonal variation. The circulating levels of endogenous steroid hormones (estrogens in females and androgens in males) are lowest in the early stages of gonadal development, increase as the gonads develop and reach maximum concentration before spawning (Sohn et al., 1999). The early stages of gonadal development (early recrudescence) occur in Fall and are dependent on the gonadotropins luteinizing hormone (LH) and follicle-stimulating hormone (FSH), which are regulated by various neurohormones, including gonadotropin-releasing hormone (GnRH). GnRH release is regulated by variety of neurohormones, including the neurotransmitter GABA (Trudeau et al., 1993a; Trudeau and Somoza, 2020). Estrogen receptors ERα, ERβI and ERβII are expressed in many tissues, including liver, gonad and brain (Choi and Habibi, 2003; Nelson et al., 2007). Estrogen receptors are present in areas of the brain with reproductive functions, such as the ventral telencephalon, preoptic area and mediobasal hypothalamus (Davis et al., 1977; Peter and Paulencu, 1980; Gelinas and Callard, 1997; Strobl-Mazzulla et al., 2008; Diotel et al., 2018) Estrogen treatment was shown to affect the production of various neurohormones including GABA in the hypothalamus in regressed male fish (Bosma et al., 2001), as well as in females (Kah et al., 1992; Pellegrini et al., 2016) leading to changes in gonadotropin production (Huggard-Nelson et al., 2002).

Androgens act through androgen receptors and can be aromatized to estrogens via aromatase, an enzyme that is present in gonad (CYP19a) (Pasmanik and Callard, 1988) and brain (Gelinas and Callard, 1997; Diotel et al., 2018). In male fish gonad, androgens are essential for gametogenesis and act on somatic cells, as androgen receptors are expressed in Sertoli and interstitial Leydig cells and affect sperm production (Schulz et al., 2010; Golshan et al., 2014, Golshan et al., 2016; Fallah et al., 2019). In midbrain, testosterone exposure increased pituitary sensitivity to GnRH in goldfish (Trudeau et al., 1993b), and can influence gonadotropin production (Habibi and Huggard, 1998) and hypothalamic cell turnover (Kinch et al., 2015). Circulating gonadotropin levels are associated with higher levels of circulating testosterone in goldfish (Sohn et al., 1999). Due to the presence of estrogen and androgen receptors in the hypothalamus (Harbott et al., 2007; Strobl-Mazzulla et al., 2008), testosterone (T) may exert an effect in the midbrain both directly and indirectly: through binding androgen receptors or via aromatization to 17ß-estradiol (E2) and subsequent estrogen receptor binding (Gelinas and Callard, 1997; Strobl-Mazzulla et al., 2008; Kinch et al., 2015).

Thyroid hormones regulate carbohydrate, lipid and cholesterol metabolic pathways and overall energy expenditure (Liu and Brent, 2010). In goldfish, thyroid hormone (3,3′,5′-Triiodo-L-thyronine (T3) in its active form) has been called a “switch” due to its role in the transition from gonadotropic to somatotropic stages in the yearly goldfish cycle (Habibi et al., 2012). An inverse association has been observed between T3 levels and circulating levels of sex steroid hormones, LH, FSH and aromatase activity (Nelson et al., 2010). T3 acts via the thyroid hormone receptor, which exhibits tissue-specific expression during the gonadal regression stage, and non-tissue specific expression during gonadal recrudescence (Nelson and Habibi, 2010). T3 was additionally found to regulate ERα, ErβI and ERβII in goldfish gonad (Nelson et al., 2010). From a seasonality perspective, circulating T3 levels are lowest right before spawn when circulating E2 and T are high (Sohn et al., 1999). There is also evidence for interaction between thyroid hormones and estrogen receptors affecting vitellogenesis and ovarian follicular development in goldfish (Nelson and Habibi, 2016).

Metabolomics methodology can provide insight into dynamic alterations to metabolism and energy allocation which occur throughout growth and reproductive phases (Ladisa et al., 2021) as well as characterize toxicometabolic responses induced by EDCs (Jordan et al., 2012; Lee et al., 2020). In real-world exposure scenarios, both terrestrial and aquatic species are exposed to mixtures of contaminants, making results from individual exposure studies less applicable in modeling the extent of physiological perturbation induced by environmental contaminant exposures. Mixture studies are important as they more closely mimic the complexity of organisms responses to contaminants, and frequently show that the response is not simply additive (Jordan et al., 2012; Kinch et al., 2015; Heys et al., 2016; Thrupp et al., 2018; Zare et al., 2018).

**FIGURE 1:**
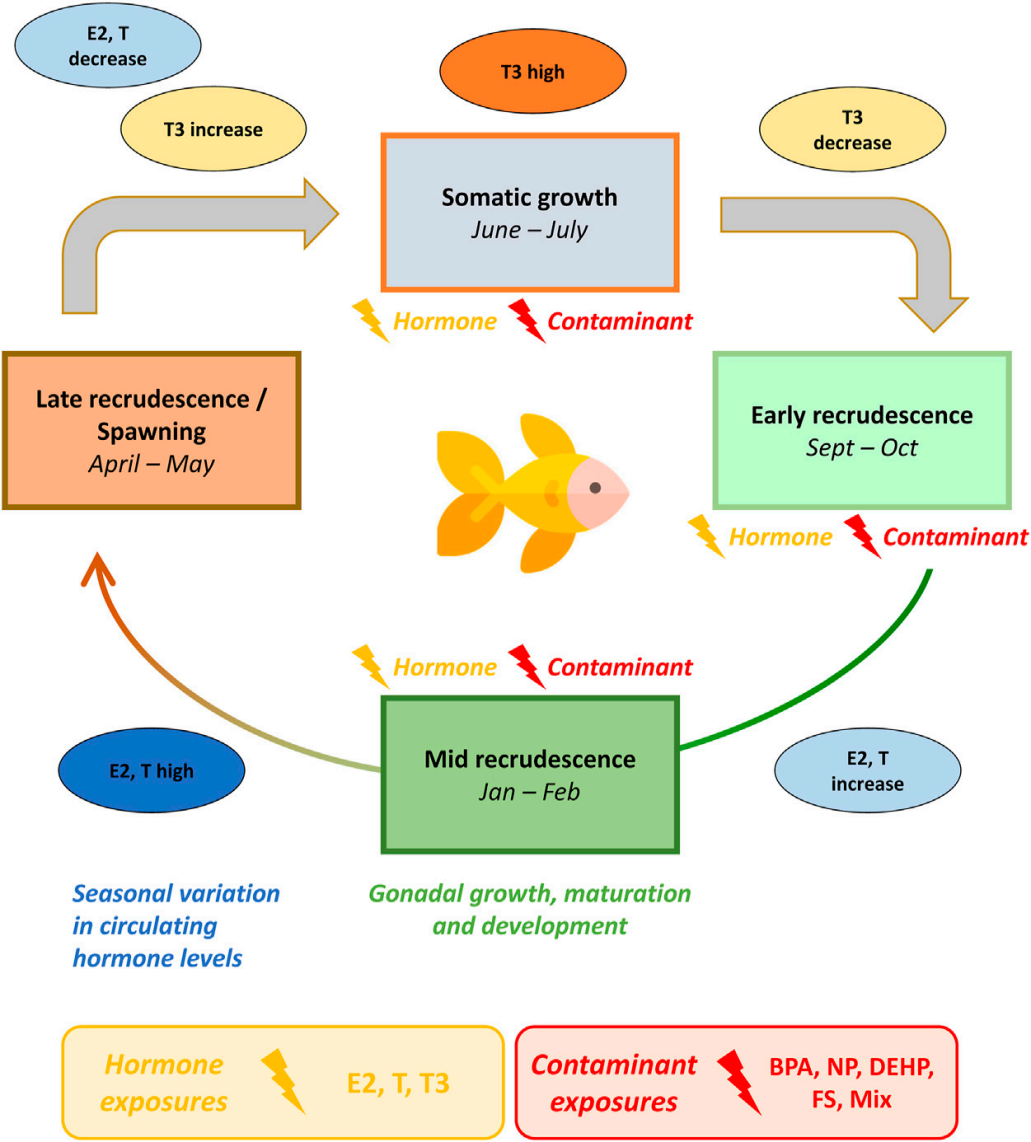
Seasonal exposure model of male goldfish to hormone and contaminant treatments across growth and reproductive stages. Goldfish are seasonal breeders with an annual reproductive cycle. Goldfish gonad starts to grow and develop in fall (early recrudescence), reaching the mid-stage of development in winter (mid-recrudescence), the late stage in spring (late-recrudescence) and spawn in summer. Endogenous hormone levels and hormone receptor expression in goldfish exhibit seasonal variation. Circulating levels of sex steroid hormones (estrogens in females and androgens in males) are lowest during early stages of gonadal development, increase as the gonads develop and reach maximum concentration before spawning. Thyroid hormone levels are high during somatic growth stages and decrease during early recrudescence. In this seasonal exposure model, male goldfish were exposed to hormones or contaminants during October (early-recrudescence), February (mid-recrudescence) and June (late-recrudescence). Abbreviations: BPA, Bisphenol A; NP, nonylphenol; DEHP, bis(2-ethylhexyl) phthalate, fucosterol; and a tertiary mixture (DEHP + NP + FS); T, Testosterone; E2, 17β-estradiol; T3, Thyroid hormone (3,3′,5′-Triiodo-L-thyronine). The goldfish icon used in this figure was made by Freepik (www.freepik.com) from www.flaticon.com.

This study was designed to assess the effects of hormones, individual contaminants and their mixture on metabolism of goldfish at different seasonal stages. Exposure effects were assessed using ^1^H-NMR metabolomics profiling of male goldfish midbrain, gonad and liver harvested during early recrudescence (October), mid-recrudescence (February) and late recrudescence (June). Compounds assessed in this study were selected based on contaminant levels reported in the Oldman and Bow Rivers in Alberta, Canada (Sosiak and Hebben, 2005) and include bisphenol A (BPA), nonylphenol (NP), bis(2-ethylhexyl) phthalate (DEHP) and fucosterol (FS). A mixture of contaminants selected based on the range of contaminants observed in the Bow River (DEHP + NP + FS) was additionally assessed (Sosiak and Hebben, 2005). Metabolome-level responses induced by contaminant exposure across tissues and seasons were benchmarked against exposure to the natural hormones E2, T and T3. Tissue and season-specific metabolic perturbations were additionally compared with responses induced by exposure to the mixture. An overview of the seasonal reproductive cycle in goldfish and the exposure paradigm used in this study is presented in Figure 1.

## Materials and Methods

### Experimental Animals

Goldfish (*Carassius auratus* ∼10 cm long, ∼30 g each) were purchased from Aquatic Imports, Calgary, Alberta. Prior to the experiment, the fish were acclimated for 72 h in flow through glass tanks (49 L) at 17°C (16–18 fish of unknown gender per tank) and fed the same amount of commercial fish food once a day (HBH Pet Products). After exposure to chemicals for 10 days, fish were sacrificed, and tissues isolated and frozen until use. All animal protocols were approved by the University of Calgary animal care committee and in accordance with the guidelines of the Canadian Council of Animal Care (protocol #AC19-0161).

### Exposure to Chemicals

17ß-estradiol (E2), 3,3′,5-Triiodo-L-thyronine (T3), testosterone (T), bis(2-ethylhexyl) phthalate (DEHP), bisphenol A (BPA), nonylphenol (NP) and fucosterol (FS) and were purchased from Sigma-Aldrich Co. Exposure concentrations used were based on measured concentrations of these chemicals in the Oldman and Bow Rivers, Alberta, Canada (Sosiak and Hebben, 2005). Experiments were run utilizing natural hormone treatments of 17ß-estradiol (500 ng/L), 3,3′,5-Triiodo-L-thyronine (500 ng/L), testosterone (500 ng/L), the Oldman river concentration for BPA (1550 ng/L) and the Bow River concentrations for DEHP (694 ng/L), NP (292 ng/L) and FS (135 ng/L). Treatments were conducted during three reproductive seasons: October (early recrudescence, i.e. onset of gonadal growth and development), February (mid-recrudescence, i.e. mid-stage of gonadal growth and development), and June (late recrudescence, i.e. late stage of gonadal development/spawn). Twenty fish were exposed in tanks treated with individual chemicals and a mixture of pollutants found in the Bow River (DEHP + NP + FS). The control group was exposed to the same concentration of the vehicle (25% DMSO; 75% EtOH). The treatment groups were randomly assigned to tanks to avoid bias. Animals were exposed for 10 days in glass aquaria supplied with activated carbon-filtered City of Calgary water (flow rate at 300 ml/min). The chemicals were added to the water every 24 h after draining the tanks to ∼10% volume and refilling them with fresh water. Therefore, animals were exposed to declining concentrations of contaminants throughout each day for 10 days. Once sacrificed, sex was determined, and liver and gonads were removed from the male goldfish for each treatment group (six to eight male fish per tank). Harvested tissues were immediately snap-frozen in liquid nitrogen and subsequently stored at −80°C until extraction and metabolomics analyses.

### Metabolite Extraction, ^1^H-NMR Spectroscopy, Data Analysis and Normalization

The metabolite extraction protocol was modified from (Atherton et al., 2008). A Bruker Advance 600 spectrometer (Bruker Biospin, Milton, Canada) was used to generate total correlation spectroscopy (2D 1H-13C TOCSY) and heteronuclear single quantum coherence spectroscopy (2D 1H-13C HSQC). Male liver and testes samples were homogenized in 2:1 methanol-chloroform solution using a tissue lyser and then sonicated in a sonication bath for 15 min. After sonication, 200 µL of a chloroform-water solution (1:1) was added to each sample and samples were then centrifuged at 13,300 rpm for 7 min at 4°C. Following centrifugation, the supernatant was transferred to a new set of 1.5 ml tubes and dried for at least 24 h using a Speedvac. Dry aqueous fractions were resuspended in 130 µL of 0.5M NaH2PO4 buffer [(DSS) = 2.5 mM in D2O, pH = 7.0]. 10 µL of 1M NaN3 was added, and the samples were vortexed for ∼15 s, pH adjusted to 7.00 if necessary, followed by the addition of 460 µL of H2O. Samples were transferred to Norell Standard series 5 mm NMR tubes for ^1^H-NMR analysis. A Bruker Advance 600 spectrometer (Bruker Biospin, Milton, Canada) with a 5 mm TXI probe at 298 K was used for ^1^H-NMR at 600.22 MHz frequency. Standard Bruker pulse sequence noesypr1d was used to obtain all one-dimensional ^1^H NMR spectra of aqueous samples and the residual water peak was irradiated during the relaxation delay of 1.0 s and during 100 ms of mixing time. 63,536 data over a spectral width of 12,195 Hz with a 90° pulse width and 5 s repetition time were acquired into 1024 scans. Prior to Fourier transformation, phasing, and baseline correction, a 0.1 Hz line broadening was applied to all the spectra. Standard Bruker pulse programs were applied to generate two-dimensional NMR experiments. The following 2D spectroscopy was performed to validate metabolite chemical shift assignments: total correlation spectroscopy (2D 1H-13C TOCSY) and heteronuclear single quantum coherence spectroscopy (2D 1H-13C HSQC).

Targeted profiling of the resulting ^1^H-NMR spectra was performed with Chenomx NMR Suite 7.5. The spectra for all samples were manually corrected for phase and baseline and then fitted with reference to the DSS peak. Metabolites were identified and quantified using the Chenomx program and its reference literature (Weljie et al., 2006) In order to ensure consistency in fitting, the spectra were fitted in random order and iteratively evaluated several times until a high degree of confidence in the consistency of the metabolite fitting was achieved. Chenomx analysis of sample spectra yielded individual metabolite concentrations utilized for further internal normalization and to account for variable sample dilutions by calculating the relative abundances of individual metabolites. A median value for each individual metabolite across all treatments was calculated, thus generating a median reference spectrum. Individual metabolite abundances were subsequently divided by the corresponding median value resulting in a “fold-change” from the median value. A new median value for the “fold changes” was calculated for each individual treatment across the metabolites. The original metabolite abundances (concentration/ion intensities) were then divided by the final median value.

### Statistical Analysis

For all experiments, multivariate statistical data analysis was performed on normalized data using SIMCA-P software. Unsupervised principal component analysis (PCA) was performed on all data to identify the most significant variances and potential outliers. Two-way orthogonal partial least squares discriminant analysis (O2PLS-DA) was utilized to assess significant variations between the treatment groups (both grouped, and pairwise O2PLS-DA models were assessed). The significance of the O2PLS-DA models was assessed based on analysis of variance testing of cross-validated predictive residuals (CV-ANOVA) in which a seven-fold cross-validation is performed during the model building process. CV-ANOVA is a significance test of a null hypothesis that the two compared models have equal cross-validatory residuals (Q2YCV) using the F distribution, and a *p*-value < 0.05 was considered significant. Specific metabolites with a Variable Influence on Projection (VIP) score >1 were deemed to be significantly altered in the multivariate O2PLS-DA models. RawGraphs 2.0 (https://app.rawgraphs.io/) was utilized for data visualization of VIP>1 metabolites from pairwise O2PLS-DA modeling and associated biochemical pathway annotation. VIP >1 metabolites and O2PLS-DA coefficients were used for Metabolite Set Enrichment Analysis (MSEA).

### Biochemical Pathway Analysis

Metabolite Set Enrichment Analysis (MSEA) was performed for all treatment-control pairs. The reason for including all treatment-control pairs was that we wanted to explore the possible effects of non-significant treatments, keeping in mind that the significance reported by O2PLS-DA is multivariate and that in non-significant cases, individual metabolites with VIP>1 may still provide insight into possible pathways implicated. A list of important compounds was entered into over representation analysis (ORA). The hypergeometric test analyzes the chances of the metabolite set repeating by chance for the compound list and provides metabolic superpathways affected with a one-sided *P*-value (Xia and Wishart, 2010).

## Results

### Grouped and Pairwise Modeling by O2PLS-DA

**FIGURE 2:**
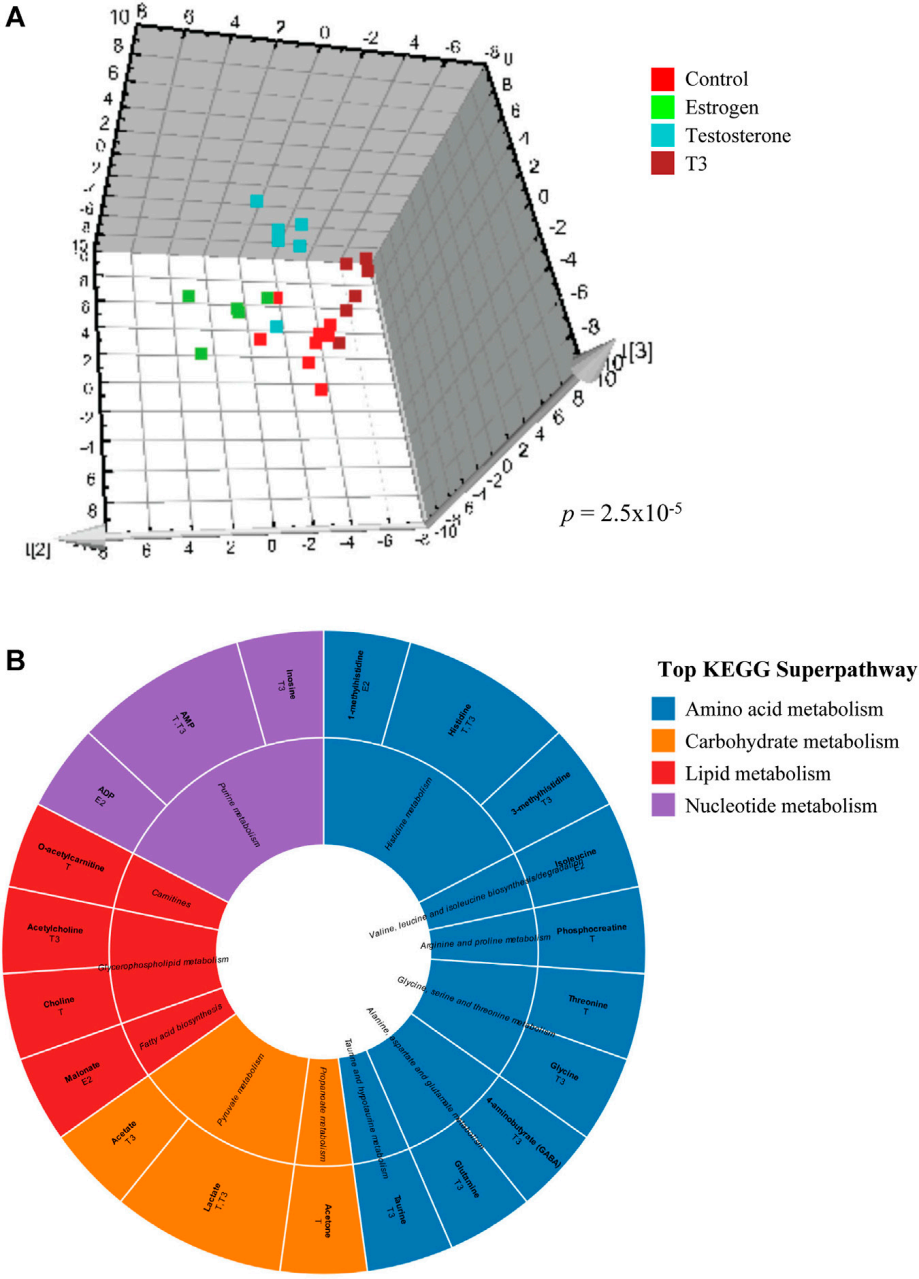
Effects of hormone treatments on midbrain metabolome of male goldfish in October. **(A)** Supervised O2PLS-DA analysis score plot illustrating the effects of control, estradiol, testosterone and T3 on midbrain metabolic profile in male goldfish treated in October. Each point represents log transformed normalized and UV scaled metabolite concentrations from ^1^H-NMR spectra. Each axis represents an orthogonal component that is a source of variation between the samples. **(B)** Sunburst diagram depicting metabolites altered by individual hormone exposures in midbrain in October. Metabolites depicted are those with VIP score >1 in pairwise control vs treatment O2PLS-DA modeling. The diagram includes annotation of specific hormone treatments found to alter each metabolite. Metabolites and associated biochemical pathways are color coded by KEGG superpathways.

Multivariate modeling by O2PLS-DA was utilized to assess metabolic impacts of hormone or contaminant treatments on goldfish midbrain, gonad and liver during early recrudescence (tissues were sampled in October), mid-recrudescence (sampled in February) and late recrudescence (sampled in June) using grouped and pairwise modeling strategies (Table 1). Grouped modeling was conducted to assess exposure-induced impacts on tissue-specific metabolomes following all treatments (hormones and contaminants), hormone treatments only and contaminant treatments only (individual contaminants + mixture) in the three seasons sampled (Table 1). A *p*-value cutoff of <0.05 was considered significant for this multivariate modeling. No grouped models containing all treatments (hormones and contaminants) were significant. One grouped model containing all hormone treatments was significant; this was observed in midbrain in October (*p 2.5e-05*) (Table 1 and Figure 2A). Grouped models assessing impact of contaminant exposures on tissue-specific metabolomes were found to be significant in midbrain in February (*p 0.036*) and in June (*p 0.042*) and in liver in June (*p 0.048*) (Table 1).

**TABLE 1:** O2PLS-DA CV-ANOVA *p* values for grouped and pairwise models for comparison of hormone, contaminant and mixture treatments in midbrain, gonad and liver across seasons.

Organ		Midbrain			Gonad			Liver		
Season		*October*	*February*	*June*	*October*	*February*	*June*	*October*	*February*	*June*
***O2PLS-DA Grouped modeling***										
All treatments (hormones and contaminants)		0.978	0.078	0.988	1.000	0.480	0.930	1.000	0.284	0.600
Hormone treatments		*2.50E-05*	0.970	0.562	0.240	0.120	0.140	0.100	0.540	0.430
Individual contaminants and mixture		0.520	*0.036*	*0.042*	0.990	0.140	0.550	1.000	0.216	*0.048*
***O2PLS-DA Pairwise modeling: Control vs individual hormone, contaminant or mixture treatment***										
**Control**	E2	1	*0.041*	0.19	0.435	*0.005*	*0.043*	1	*0.023*	*0.042*
	T	0.055	*0.033*	*0.043*	0.425	*0.012*	*0.023*	1	*0.0367*	0.48
	T3	*0.019*	*0.031*	*0.035*	*0.005*	*0.037*	0.061	*0.035*	*0.034*	*0.031*
	BPA	*0.0186*	*0.0016*	-	0.142	*0.0015*	-	0.684	*0.0167*	-
	Mix	0.12	*0.0081*	*0.0269*	0.45	*0.033*	0.57	0.127	*0.034*	0.34
	NP	0.91	*0.0031*	0.157	*0.036*	*0.0138*	0.13	0.262	*0.027*	0.165
	FS	1	*2.24E-06*	*0.026*	0.7	*0.029*	0.16	0.115	*0.0231*	0.072
	DEHP	0.87	*0.0078*	*0.016*	0.624	*0.02*	0.14	0.06	*0.0019*	0.02
***O2PLS-DA Pairwise modeling: Hormone vs contaminant treatment***										
**Estradiol - contaminant comparison**										
**E2**	BPA	0.273	*0.029*	-	*0.0137*	*0.026*	-	0.54	0.054	-
	NP	0.515	*0.014*	1	*0.029*	*0.018*	0.13	1	*0.027*	0.37
	FS	0.448	*0.012*	*0.0194*	0.088	*0.0214*	0.09	0.74	0.083	0.057
	DEHP	0.22	*0.035*	*0.0478*	*0.048*	*0.045*	0.22	1	*0.0088*	0.023
**Testosterone - contaminant comparison**										
**T**	BPA	*0.0022*	*8.30E-05*	-	*0.01*	*0.0076*	-	1	0.284	-
	NP	0.058	*4.80E-04*	0.11	*0.025*	*0.0084*	0.062	*0.046*	*0.021*	0.35
	FS	1	*7.62E-05*	*4.60E-05*	0.136	*0.028*	*3.40E-04*	1	0.14	*0.047*
	DEHP	*0.014*	*0.0061*	*0.0014*	0.283	*0.0189*	0.096	0.67	*0.036*	0.12
**T3 - contaminant comparison**										
**T3**	BPA	0.235	*0.0021*	-	*0.034*	*0.029*	-	1	*0.047*	-
	NP	1	*9.40E-04*	0.08	*0.03*	*0.033*	*0.046*	1	0.72	*0.018*
	FS	*2.00E-04*	0.36	*1.40E-04*	0.142	*0.054*	*0.015*	1	*0.036*	*0.008*
	DEHP	*0.033*	*0.032*	*2.00E-03*	*0.03*	*0.042*	*0.039*	0.97	0.112	0.075
***O2PLS-DA Pairwise modeling: Mixture vs individual components***										
**Mixture - contaminant comparison**										
**Mix**	NP	0.152	1	1	0.455	1	0.32	1	1	*0.007*
	FS	*0.023*	0.42	0.133	1	0.24	*0.014*	*0.0435*	1	0.083
	DEHP	1	1	1	0.09	1	0.64	0.56	1	0.22

Values in italics indicate that the p value is significant.

**FIGURE 3:**
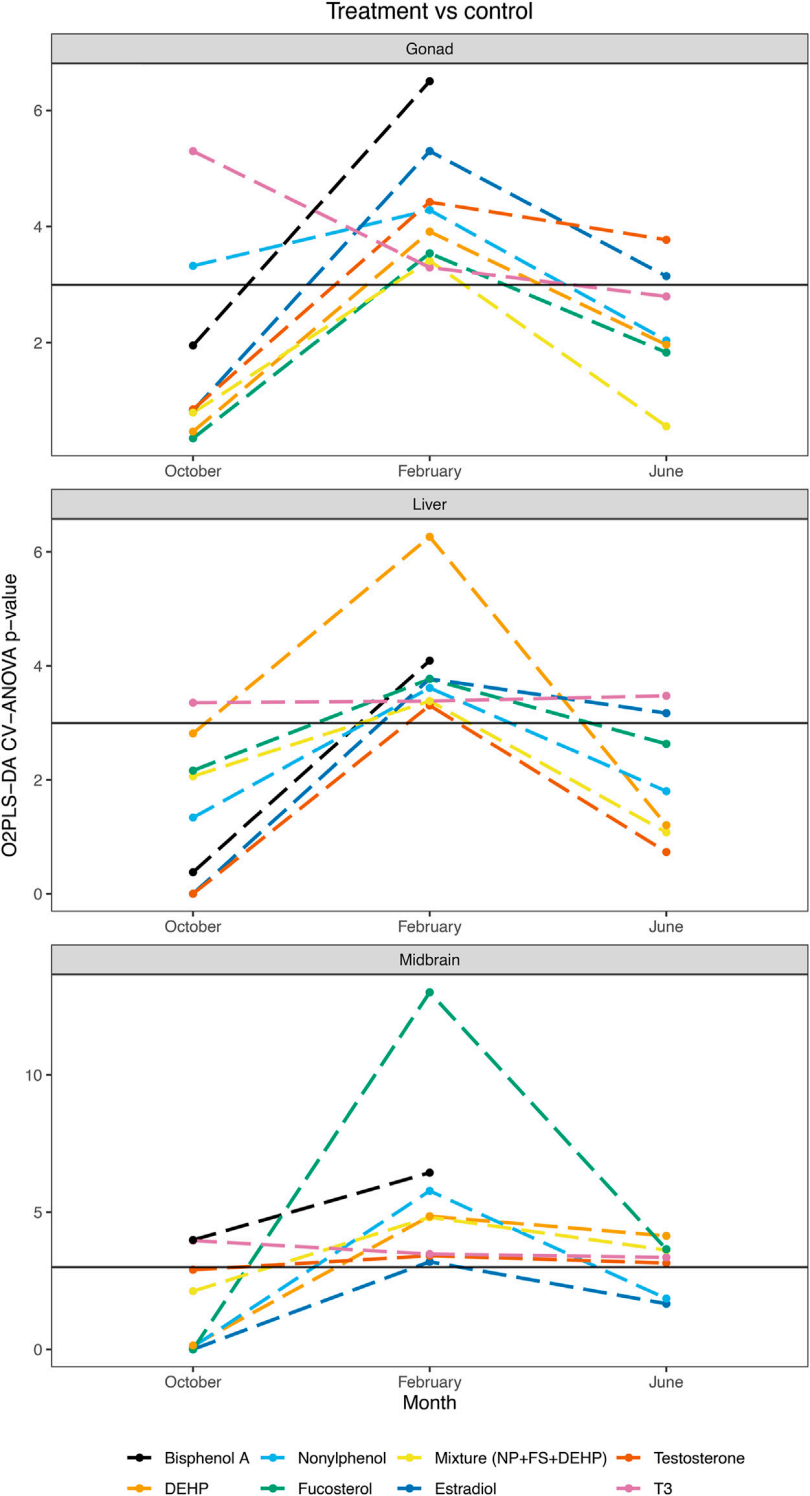
O2PLS-DA control vs treatment pairwise modeling summary across tissues and seasons. Summary of O2PLS-DA pairwise modeling results assessing control vs individual treatment pairs in midbrain, gonad and liver in October, February and June. CV-ANOVA *p*-values are plotted on a logarithmic scale.

A pairwise O2PLS-DA modeling strategy was additionally employed in order to assess specific impacts of individual hormones or contaminants on midbrain, gonad and liver metabolomes at different stages of reproductive development (Table 1). When control vs each treatment (hormone, contaminant, or mixture of contaminants) was modeled across tissues and seasons, a clear seasonal dependence of exposure-induced metabolic changes was observed (Table 1 and Figure 3). In October, select control—treatment pairs resulted in significant pairwise O2PLS-DA models, including T3 in midbrain, gonad and liver, BPA in midbrain and NP in gonad (Table 1 and Figure 3). In February, all control—treatment pairs resulted in significant O2PLS-DA models in midbrain, gonad and liver (Table 1 and Figure 3). In June, several hormone exposures resulted in significant pairwise models across tissues (E2 in gonad and liver; T in midbrain and gonad; and T3 in midbrain and liver). Several contaminant treatments significantly altered the midbrain metabolome in June, including FS, DEHP and mixture. Individual contaminant and mixture treatments did not result in significant pairwise O2PLS-DA models in gonad or liver in June (Table 1 and Figure 3). Figure 3 provides a summary of pairwise modeling results (logP value of control—treatment pairs assessed by O2PLS-DA) across tissues and seasons.

Further pairwise multivariate modeling was conducted to benchmark metabolome-level alterations induced by contaminant exposures against responses observed following exposure to the natural hormones, and to compare metabolome-level changes induced by a contaminant mixture as compared with its individual components (Table 1). Similar to pairwise modeling conducted on control—treatment pairs, a seasonal dependence was observed in O2PLS-DA models assessing contaminant vs hormone treatments (Table 1 and Supplementary Figure 1). In midbrain, all hormone—contaminant comparisons assessed were found to be significant in February, with the exception of T3 vs FS (*ns*). In gonad in February, all hormone—contaminant comparisons assessed were found to be significant, and in liver half of the hormone—contaminant comparisons generated significant models in February. Across tissues, fewer pairwise models assessing hormone—contaminant comparisons were significant in October and in June (Table 1 and Supplementary Figure 1). When comparing metabolic changes induced by mixture treatment as compared with its individual components, few pairwise O2PLS-DA models were found to be significant. These included FS vs mixture treatment in midbrain and liver in October, and in gonad in June, and NP vs mixture treatment in liver in June (Table 1 and Supplementary Figure 1). Supplementary Figure 1 provides a summary of pairwise modeling results (logP value of endogenous hormone—individual contaminant pairs assessed by O2PLS-DA) across tissues and seasons.

### Data Analysis and Visualization Strategy

In the O2PLS-DA multivariate models, specific metabolites with a VIP score >1 were considered significantly altered by exposure. Two main approaches were subsequently utilized for visualizing metabolome-level changes induced by hormone and contaminant treatments across tissues and seasons. The first strategy was to manually annotate all VIP metabolites identified by multivariate modeling with the most relevant KEGG pathways and superpathways associated with the metabolite. The purpose of this annotation was to assess and visualize the impact of metabolite-level alterations induced by hormone and contaminant exposures on major metabolic processes. Supplementary Table 1 details the annotation of study VIP metabolites with their associated KEGG pathways and indicates in which tissue(s) each metabolite was found to be altered. For each VIP metabolite, a top KEGG metabolic pathway and top KEGG metabolic superpathway was selected and subsequently utilized for data visualization. This allowed for visualization of hormone and contaminant induced changes at the metabolite level and assessment of impact on major categories of metabolism. The second strategy was to conduct a metabolite set enrichment analysis (MSEA) to assess biochemical pathways significantly enriched by hormone or contaminant exposure across tissues and seasons.

Grouped O2PLS-DA modeling results (Table 1) were utilized as a starting point for identifying windows of vulnerability to metabolic perturbation following hormone or contaminant exposure in male goldfish at different stages of gonadal recrudescence. Grouped modeling revealed vulnerability of male goldfish midbrain to hormone exposures in October (*CV-ANOVA p 2.5e-05*), and to contaminant and mixture exposures in February (*CV-ANOVA p 0.036*) and in June (*CV-ANOVA p 0.042*). An additional point of vulnerability was observed in male goldfish liver following contaminant and mixture exposures in June (*CV-ANOVA p 0.048*) (Table 1). In order to take a closer look at model components that may be driving the overall vulnerability of male goldfish to hormone and contaminant exposures, a pairwise modeling strategy was subsequently employed. Pairwise O2PLS-DA models assessing each control—treatment pair (models summarized in Table 1) were utilized for in-depth analysis and visualization of metabolic alterations induced by hormone or contaminant treatments in each tissue and season.

### Metabolite-Level Impacts of Hormone and Contaminant Treatments Across Tissues and Seasons

A representative grouped O2PLS-DA analysis score plot is shown in Figure 2A, depicting the effects of hormone treatments (estradiol, testosterone and T3) on metabolic profiles of male goldfish in October (early recrudescence) (*CV-ANOVA p 2.5e-05*). Each point represents log transformed normalized and UV scaled metabolite concentrations from ^1^H-NMR spectra and each axis represents an orthogonal component that is a source of variation between the samples. Individual metabolites altered by hormone treatments in midbrain in October are shown in Figure 2B, with annotation of the major KEGG pathways and superpathways associated with these metabolites.

**FIGURE 4:**
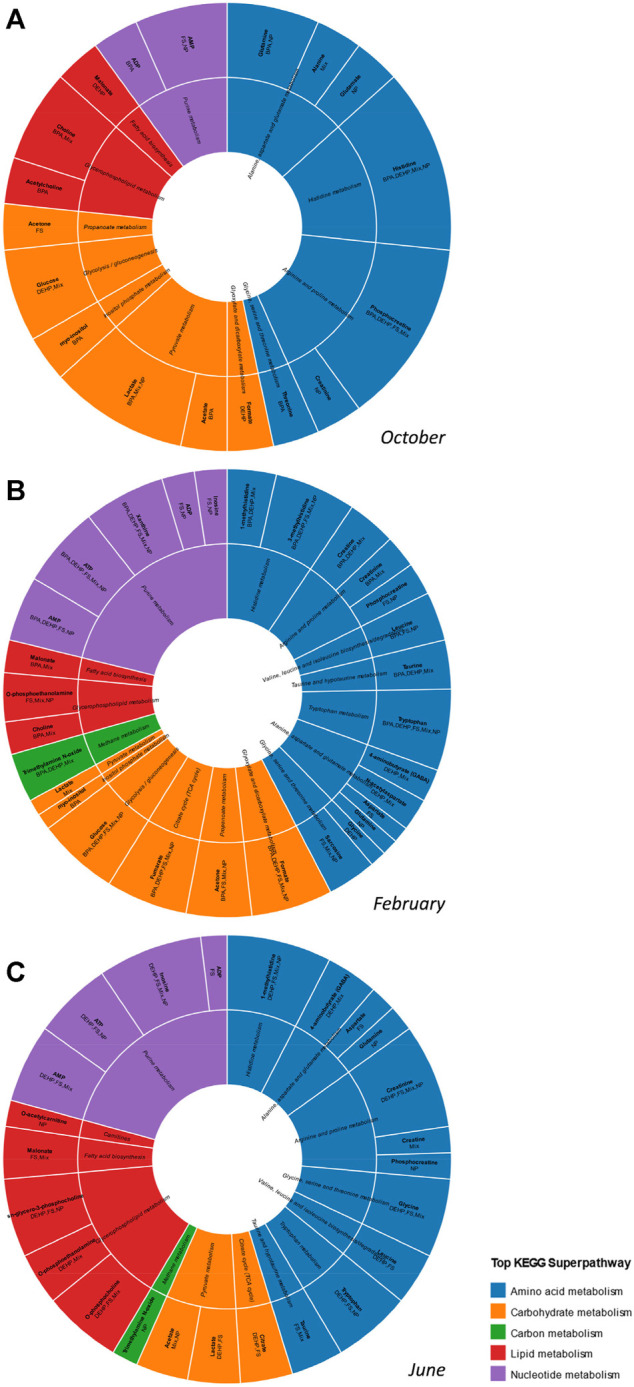
Effects of contaminant treatments on male goldfish midbrain metabolome across seasons. Sunburst diagram depicting metabolites altered by individual contaminants or the tertiary mixture in midbrain in **(A)** October **(B)** February and **(C)** June. Metabolites depicted are those with VIP score >1 in pairwise control vs treatment O2PLS-DA modeling. The diagram includes annotation of specific contaminant treatments found to alter each metabolite. Metabolites and associated biochemical pathways are color coded by KEGG superpathways.

Figure 4 depicts metabolites altered by contaminant exposure in midbrain across seasons, characterized according to KEGG pathways. A common signature of alteration to carbohydrate-related metabolites is observed in midbrain in October and February with less of an impact on this class of metabolites in June. A proportionally greater impact on purines/purine metabolism is observed in February and June as compared with October, and lipid-related metabolites were most altered in midbrain in June. A high proportion of amino acids/amino acid metabolism-related metabolites were altered across all seasons and the overall number of contaminant-altered metabolites was greatest in February (Figure 4).

June was observed to be a period of enhanced vulnerability to contaminant-induced metabolic perturbation in male goldfish liver (Table 1; *CV-ANOVA p 0.048*). Metabolites perturbed by contaminant or mixture exposure in liver in June are detailed in Supplementary Figure 2. Supplementary Table 2 provides a complete list of VIP metabolites identified across all pairwise multivariate datasets (control—treatment pairs) with indication of tissue and season in which the metabolite was altered and direction of change.

### Metabolite Set Enrichment Analysis Following Hormone and Contaminant Treatments Across Tissues and Seasons

**FIGURE 5:**
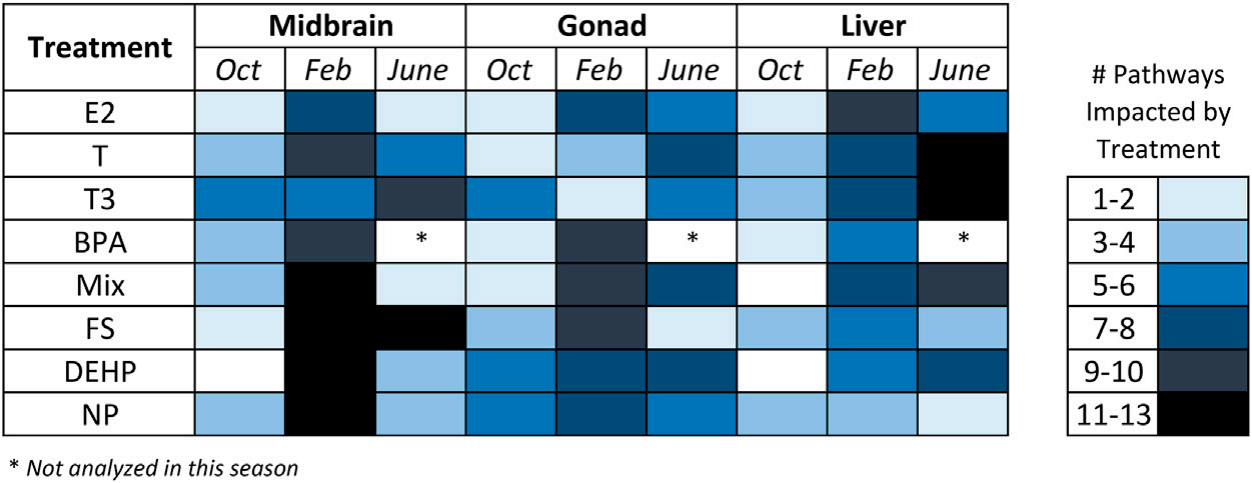
Number of biochemical pathways altered by hormone, contaminant or mixture treatments in midbrain, gonad and liver. Heatmap depicting the number of biochemical pathways significantly altered by individual hormone or contaminant exposures across tissues and seasons analyzed. Results are based on the Metabolite Set Enrichment Analysis (MSEA). Abbreviations: BPA, Bisphenol A; NP, nonylphenol; DEHP, bis(2-ethylhexyl) phthalate, fucosterol; and a tertiary mixture (DEHP + NP + FS); T, Testosterone; E2, 17β-estradiol; T3, Thyroid hormone (3,3′,5′-Triiodo-L-thyronine).

**FIGURE 6:**
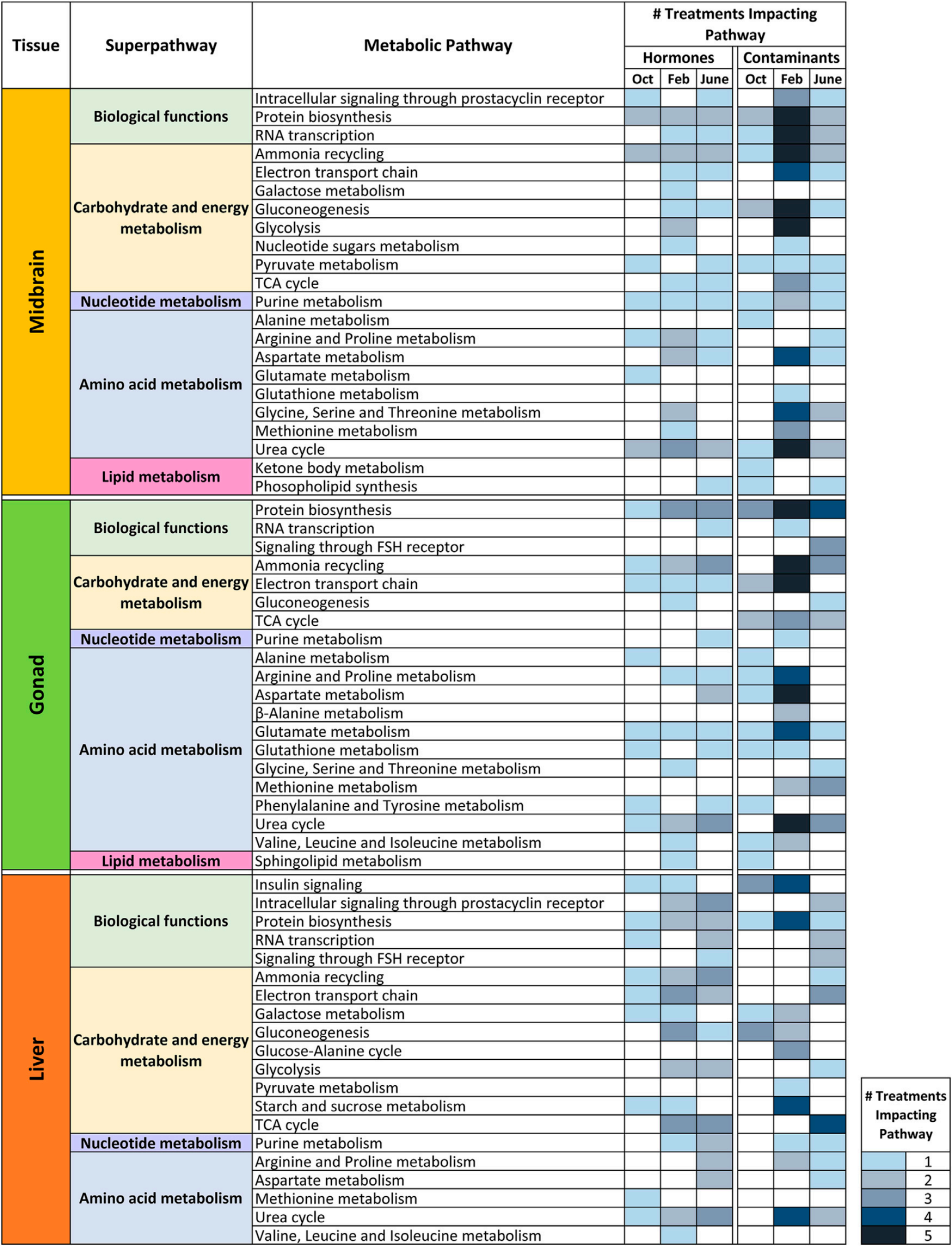
Impact of hormone or contaminant treatments on biochemical pathways in midbrain, gonad and liver in October, February and June. Heatmap depicting a summary-level view of specific biochemical pathways altered by hormone or contaminant exposures across tissues and seasons analyzed. Biochemical pathways are categorized by KEGG superpathways. The graded-color heatmap depicts the number of treatments found to impact a biochemical pathway in a particular tissue and season. Results are based on the Metabolite Set Enrichment Analysis (MSEA).

Metabolite set enrichment analysis (MSEA) was conducted for all control—treatment pairs and MSEA results with a *p* value <0.05 were considered significant. For this analysis, VIP>1 metabolites from all pairwise (control—treatment) O2PLS-DA models were included in the over-representation analysis regardless of whether the overall model was significant. The reason for this was to have a broad scope for examining metabolite-level changes and biochemical pathways that may be impacted by hormone and contaminant exposures across tissues and seasons. Figures 5, 6 and Supplementary Figure 3 summarize MSEA results across tissues and seasons following hormone and contaminant exposures. Figure 5 summarizes the number of biochemical pathways significantly altered by hormone or contaminant exposure across tissues and seasons analyzed, with number of pathways altered sorted by treatment (hormone or control). Across tissues, February is found to be a period of enhanced vulnerability to metabolic disruption following both hormone and contaminant exposures. Some treatment-specific windows of vulnerability are additionally observed, for example T and T3 perturbed >11 biochemical pathways in liver in June, and FS perturbed >11 biochemical pathways in midbrain in June. Figure 6 presents a summary-level view of biochemical pathways significantly altered by individual hormone, contaminant or mixture exposures across tissues and seasons. The heatmap (graded blue shading) depicts the number of hormone or contaminant treatments found to alter a particular biochemical pathway and indicates the tissue and season in which this occurred. Supplementary Figure 3 presents an expanded view of biochemical pathway alterations induced by hormone or contaminant exposures. The black shading indicates biochemical pathways altered by specific hormone and contaminant treatments in each tissue and season. As observed with the pairwise O2PLS-DA modeling, a clear window of enhanced vulnerability to metabolic disruption following contaminant exposure was observed across tissues in February. June was second in terms of overall vulnerability to metabolic perturbation following hormone or contaminant exposures. Biochemical pathways found to be altered by the MSEA analysis across the datasets include carbohydrate and energy metabolism related pathways, purine metabolism, a range of amino acid metabolism related pathways and select lipid metabolism related pathways (Figure 6).

In midbrain, superpathways most affected overall include carbohydrate and energy metabolism, as well as amino acid metabolism related pathways (Figure 6). E2 exerted its greatest impact on metabolism in February. Pathways altered by E2 include glycolysis and gluconeogenesis, protein biosynthesis and several amino acid metabolism-related pathways. In midbrain, T exerted its greatest impact in February; most of these alterations were related to carbohydrate and energy metabolism. T3 altered the greatest number of biochemical pathways in June, and these pathways were predominantly related to carbohydrate and energy metabolism. Ammonia recycling and urea cycle were altered across hormone exposures, by E2 in February only and by T and T3 in most seasons. Following contaminant exposures in midbrain, the greatest biochemical impacts were observed in February for all contaminants tested (Figure 6 and Supplementary Figure 3). Pathways most commonly altered in midbrain by contaminant exposures include RNA transcription, ammonia recycling, gluconeogenesis, glycolysis and amino acid metabolism related pathways including aspartate metabolism, glycine, serine and threonine metabolism and urea cycle.

In gonad, amino acid metabolism was most affected overall by hormone and contaminant exposures across seasons (Figure 6). E2 was observed to exert its greatest impact on gonad metabolism in February, while T exerted its greatest impact on gonad metabolism in June. T3 altered a variety of biochemical pathways in both October and June. Contaminants exerted their greatest effect on gonad metabolism in February. The most commonly impacted pathways following contaminant exposures in gonad in February include ammonia recycling and electron transport chain (carbohydrate and energy metabolism superpathway) as well as amino acid metabolism-related pathways arginine and proline metabolism, aspartate metabolism, glutamate metabolism and urea cycle (Figure 6 and Supplementary Figure 3). Several contaminant exposures additionally altered protein biosynthesis in June. The tertiary mixture exposure altered a greater number of biochemical pathways in gonad in June than any of its individual components.

**FIGURE 7:**
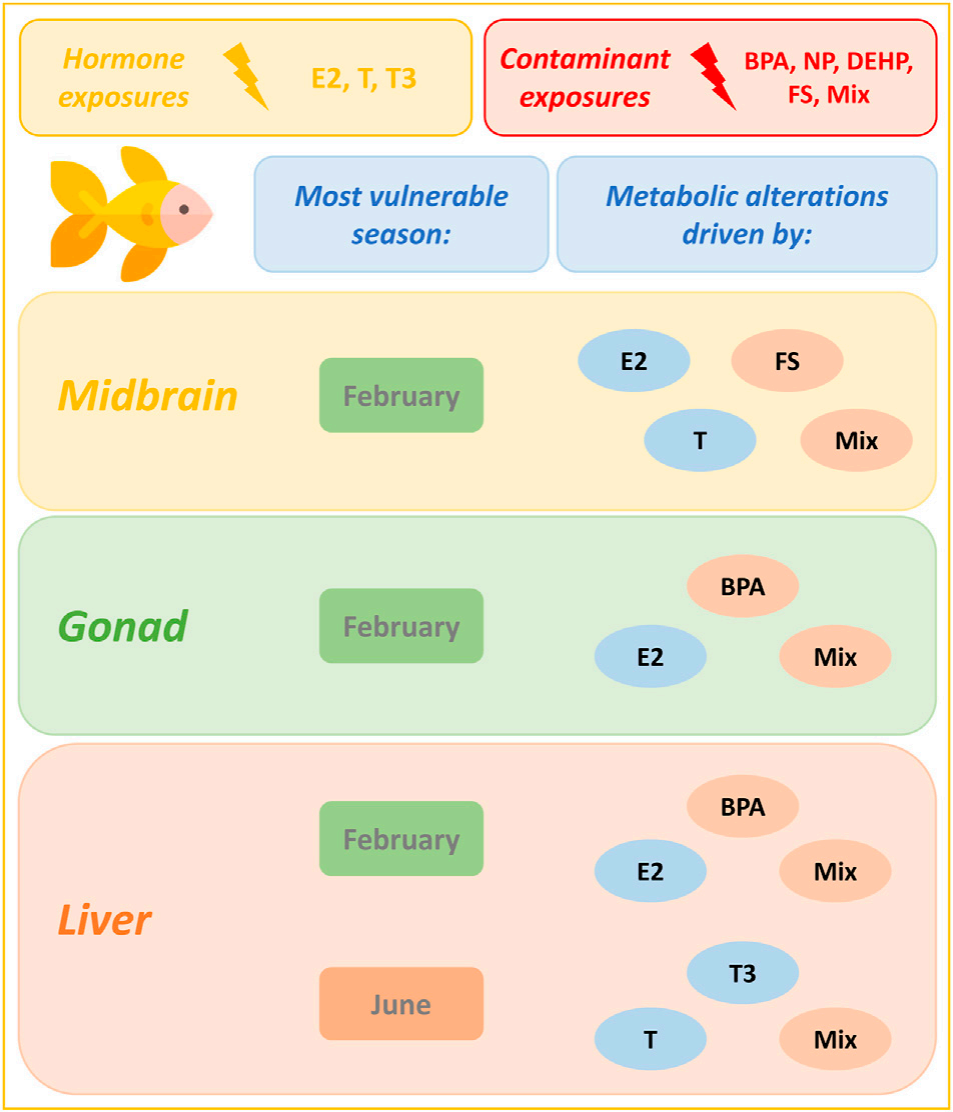
Seasonal dependence of male goldfish to metabolic alterations induced by hormone and contaminant exposures. This figure models seasonal dependence of male goldfish to metabolic alterations induced by hormone and contaminant exposures across growth and reproductive stages. In all tissues analyzed, February is observed to be a period of enhanced vulnerability to metabolic alteration. An additional window of susceptibility is observed in liver in June. Hormones and contaminants contributing most extensively to biochemical pathway alterations in a particular tissue and season are highlighted. Results are based on the Metabolite Set Enrichment Analysis (MSEA). The goldfish icon used in this figure was made by Freepik (www.freepik.com) from www.flaticon.com.

In liver, carbohydrate and energy metabolism was most affected overall by hormone and contaminant exposures (Figure 6). E2 exerted its greatest impact on liver biochemical pathways in February, while T and T3 exerted their greatest impact in June. Male goldfish liver appears to be equally vulnerable to metabolic disruption from hormone and contaminant exposures in February and in June (Figure 6 and Supplementary Figure 3). Pathways most commonly altered by contaminants in February include insulin signaling, protein biosynthesis, starch and sucrose metabolism and urea cycle. Pathways most commonly altered by contaminant exposures in June include TCA cycle and electron transport chain. In June, hormones (driven by T and T3 exposures) perturbed a wide range of liver biochemical pathways. As observed in gonad, the tertiary mixture in June induced a greater impact on liver biochemical pathways than any of its individual components. Figure 7 summarizes windows of vulnerability to hormone and contaminant exposures in male goldfish and indicates hormone and contaminant exposures observed to have the greatest impact on metabolism across growth and reproductive stages of male goldfish.

## Discussion

### Toxicometabolomics Approach to Assess Windows of Vulnerability to Metabolic Disruption by EDCs Across Growth and Reproductive Stages of Male Goldfish

In this study, we have explored seasonal responses in male goldfish to metabolic perturbation in the presence of endocrine disruptors as well as natural hormones in midbrain, gonad and liver tissues. Using an O2PLS-DA modeling strategy, grouped modeling of hormone or contaminant exposures indicated periods of enhanced vulnerability to hormone or contaminant exposure in select tissues and seasons, including midbrain following hormone exposure in October, midbrain following contaminant exposure in February and June, and liver following contaminant exposure in June (Table 1). The control—treatment pairwise O2PLS-DA modeling was more informative overall as it allowed for metabolome-level changes induced by each hormone or contaminant treatment to be examined in each tissue and season. This pairwise modeling was utilized as the basis for examining metabolite-level changes and conducting biochemical pathways modeling across datasets.

Metabolic alterations induced by hormone or contaminant exposures in male goldfish midbrain, gonad and liver exhibited a clear seasonal dependence. In February (mid-recrudescence stage), goldfish were most vulnerable to metabolic perturbation induced by hormone or contaminant exposures, and during this stage, metabolome-level responses induced by contaminant exposures were most different from responses induced by the natural hormones (Table 1). Metabolites determined to be significantly altered in any of the datasets (VIP>1 metabolites) were annotated with the most relevant KEGG metabolic pathways (Supplementary Table 1). While top sub- and superpathways were selected for the purpose of data visualization (Figure 2, Figure 4 and Supplementary Figure 2), it is worth noting that many of the VIP metabolites detected can feed into a variety of different metabolic pathways, for example glycine and malonate, which are involved in both amino acid metabolism as well as lipid metabolism related pathways and the amino acids glutamate and glutamine which function as neurotransmitters. This is also the case with nucleotides such as ADP, which is involved in purine metabolism (nucleotide metabolism superpathway), oxidative phosphorylation (energy metabolism superpathway) as well as amino sugar and nucleotide sugar metabolism (carbohydrate metabolism superpathway) (Supplementary Table 1). When examining biochemical pathways altered by hormone and contaminant exposures across tissues and seasons, a broad scope was taken in which VIP metabolites from all O2PLS-DA control-treatment pairwise models (both significant and non-significant) were examined in the over-representation analysis.

### Endogenous Hormone Treatments Alter Midbrain, Gonad and Liver Metabolic Homeostasis in a Season-dependent Manner

E2, T and T3 play a vital role in regulating the reproductive cycle in goldfish (Figure 1) (Sohn et al., 1999; Nelson et al., 2010). In this study, the effects of these endogenous hormones on midbrain, gonad and liver metabolism were observed to have seasonal dependence. In midbrain, response to E2 and T exhibited seasonal variation (Table 1) and in the MSEA analysis, E2 and T exerted their greatest biochemical pathway impact in February (Supplementary Figure 3). Some pathways were commonly altered by E2 and T treatments in midbrain in February, such as glycolysis, aspartate metabolism and urea cycle. The remainder of the specific pathway impacts were unique to either E2 or T exposure. In male goldfish midbrain, it’s unclear whether effects of T across seasons are mediated via AR or ER, after aromatization. Thyroid hormone (T3) significantly altered midbrain metabolism in all three seasons (Table 1). A range of biochemical pathway alterations induced by T3 were observed across the three seasons and in June, biochemical pathways impacts were largely related to carbohydrate and energy metabolism (Supplementary Figure 3).

Mammalian studies suggest that estrogen, signaling through ERs, plays an important role in gonadal development and function (Akingbemi, 2005). In male goldfish, E2 regulates expression of gonadal ER subtypes (Nelson et al., 2007; Marlatt et al., 2008) and in a rodent model, E2 altered steroidogenesis in testicular Leydig cells (Lee et al., 2012). T plays a critical role in spermatogenesis and steroidogenesis in male gonad, and ARs are expressed in Sertoli and interstitial cells in teleost fish gonad (Schulz et al., 2010). T3 was found to regulate ERs in goldfish gonads (Nelson et al., 2010). In this study, E2, T and T3 significantly altered metabolism in male goldfish gonad in a season-dependent manner (Table 1). Overall biochemical pathways alterations in gonad were most driven by E2 exposure in February, and by both T and T3 exposures in June (Supplementary Figure 3). Pathways most commonly affected by hormone exposures in gonad include protein biosynthesis, ammonia recycling and urea cycle (Figure 6).

In liver, E2 plays a well-established role in regulating vitellogenin (Vtg) production (Choi and Habibi, 2003; Nelson et al., 2007; Nelson and Habibi, 2010). Vtg production can be induced in male fish due to treatment with exogenous estrogen (Soverchia et al., 2005; Nelson and Habibi, 2016) and this is considered to be a sign of estrogenic endocrine disruption in males. Thyroid hormone affects carbohydrate, lipid and cholesterol metabolism and regulates energy expenditure (Liu and Brent, 2010). In goldfish it also acts as a “switch” towards somatic growth (Habibi et al., 2012). In liver, E2 and T alter metabolism in a seasonally-dependent manner, while T3 significantly affected metabolism in all three seasons (Table 1). Biochemical pathway impacts in liver following hormone exposures were driven by E2 in February, and by both T and T3 in Feb and June (Supplementary Figure 3 and Figure 7). Biochemical pathways most commonly affected in liver were related to carbohydrate and energy metabolism.

### Individual Contaminant Treatments and the Tertiary Mixture Perturb Midbrain, Gonad and Liver Metabolism in a Season-dependent Manner

We have previously observed perturbations to male goldfish liver metabolism following EDC exposure alone and in mixture (Jordan et al., 2012). Liver transcriptomics studies have shed light on alterations to lipid metabolism and the hepatic transcriptome following EDC exposure (Santangeli et al., 2018; Zare et al., 2018). EDC alter neurotransmitter receptor pathways in brain as well as overall gene expression patterns (Martyniuk et al., 2006; Gore, 2010) and polychlorinated biphenyls were observed to affect GnRH gene expression in GT-1 cell lines (Gore, 2002). In gonad, BPA exhibits estrogenic and anti-androgenic properties (Takayanagi et al., 2006; Hatef et al., 2012). BPA exposure in male goldfish adversely impacted male gonad physiology and sperm motility (Hatef et al., 2012). In zebrafish embryo, BPA was shown to affect hypothalamic turnover, leading to hyperactivity via mechanisms involving AR and aromatase activity (Kinch et al., 2015). NP exposure resulted in an increase of apoptosis in teleost fish gonad (Kaptaner and Ünal, 2011; Sayed et al., 2012). DEHP increased apoptosis in a rodent fetal testis cells model (Muczynski et al., 2012) and exhibited anti-androgenic effects in cultured human testis lines (Desdoits-Lethimonier et al., 2012). FS has not been studied as extensively, but was determined to incite cytotoxicity (Khanavi et al., 2012; Permeh et al., 2012). In liver, EDCs interact with a variety of hormonal and nutrient sensing receptors (Zizola et al., 2008; Feige et al., 2010; Kassotis and Stapleton, 2019). BPA and NP are additionally known to have estrogenic properties on male liver, due to their induction of Vtg (Soverchia et al., 2005; Hatef et al., 2012).

In the present study, a range of EDCs and their tertiary mixture induce seasonally specific alterations to metabolism in midbrain, gonad and liver of male goldfish. In midbrain, February was observed to be the season of greatest vulnerability to metabolic perturbation following contaminant exposures (Table 1). FS, DEHP and Mixture additionally altered the midbrain metabolome in June, and only BPA significantly altered the midbrain metabolome in October (Table 1). FS was a major driver of midbrain biochemical pathway alterations, with a range of biochemical pathways altered in February and June (Supplementary Figure 3). In February, FS as well as mixture exposures altered the greatest number of biochemical pathways (Supplementary Figure 3 and Figure 7). In gonad, contaminants exerted an overall greater impact on gonad biochemical pathways than hormones, and February was observed to be the season most vulnerable to metabolic perturbation (Table 1 and Figure 6). Biochemical pathway alterations in gonad appeared to be driven largely by BPA and mixture exposures in February, and by mixture exposure in June (Supplementary Figure 3 and Figure 7). Contaminant treatments significantly altered the liver metabolome in February only based on results from pairwise O2PLS-DA modeling (Table 1). Biochemical pathways modeling (MSEA) revealed a more sparse distribution of pathways effects induced by contaminant treatments in liver compared with those observed in midbrain and gonad across seasons. Among contaminant exposures in liver, the tertiary mixture altered the greatest number of biochemical pathways in both February and June (Supplementary Figure 3 and Figure 7).

### Metabolic Responses Induced by Contaminants Differ From Responses Induced by Endogenous Hormones in a Season-dependent Manner

Individual contaminants tested in this study (BPA, NP and DEHP) are known for their versatile interaction with hormonal and nutrient sensing receptors (Kwak et al., 2001; Lovekamp-Swan et al., 2003; Kwintkiewicz et al., 2010; Hatef et al., 2012; Kassotis and Stapleton, 2019) and cytotoxicity in case of fucosterol (Khanavi et al., 2012). Strong interactions with estrogen and androgen systems were demonstrated for BPA (Hatef et al., 2012; Kinch et al., 2015) and NP (Lee et al., 2003). DEHP interacts with androgenic pathways (Desdoits-Lethimonier et al., 2012), as well as with estrogenic systems at higher concentrations (Uren-Webster et al., 2010). BPA, NP and DEHP have been found to interact with thyroid hormone signaling (Ghisari and Bonefeld-Jorgensen, 2009). When comparing metabolome-level responses induced by contaminants against those induced by endogenous hormones (O2PLS-DA pairwise modeling), seasonal variation in response was observed. Contaminant-induced responses differed from those induced by endogenous hormones most frequently in February overall, and exhibited additional tissue and season specificity (Table 1). In midbrain and gonad, contaminants frequently induced exposure effects that could be differentiated from those induced by hormones across the three seasons, while comparisons of contaminant vs hormone responses in liver exhibited less significance overall (Table 1).

### Individual Contaminant Treatment Comparison With Tertiary Mixture

Previous studies have demonstrated that mixtures of contaminants exert effects that are not simply additive when compared with those induced by their individual components (Jordan et al., 2012; Kinch et al., 2016; Thrupp et al., 2018; Zare et al., 2018). In this study, the tertiary mixture of nonylphenol, fucosterol and DEHP had a significant global metabolic effect on midbrain tissue in February and June and on liver and gonad tissues in February only. When pairwise O2PLS-DA modeling was conducted to compare metabolome-level alterations induced by mixture in each tissue and season with those induced by individual components, only select comparisons resulted in a significant pairwise model (Table 1). These included FS vs mixture exposure in midbrain and liver in October and in gonad in June, and NP vs mixture exposure in liver in June (Table 1). At the level of biochemical pathways modeling (conducted using MSEA), the mixture frequently impacted the greatest number of biochemical pathways during seasons of heightened vulnerability to metabolic perturbation; this was observed in all three tissues in February and additionally in liver in June (Supplementary Figure 3 and Figure 7). We have previously demonstrated gain of function of mixture when assessing impact of environmental contaminants on goldfish liver metabolome using NMR metabolomics profiling (Jordan et al., 2012). Expanding this approach to profile multiple tissues across different growth and reproductive stages indicates a potential gain of function of mixture exposure at the level of biochemical pathways modeling when examining tissue and season-specific exposure impacts.

## Conclusion

In this study, we have examined toxicometabolomic responses to EDCs alone and in mixture across growth and reproductive stages of male goldfish. Study strengths include profiling organ-specific metabolome-level responses across seasons, benchmarking EDC-induced responses against metabolic effects of endogenous hormone treatment, and examining responses induced by exposure to individual EDCs compared with those induced by a tertiary mixture. We observe a clear seasonal dependence to metabolome-level alteration induced by hormone or contaminant exposures, with February (mid-recrudescence) the stage at which male goldfish are most vulnerable to metabolic perturbation. Comparisons of metabolome-level responses induced by contaminants against those induced by endogenous hormones also exhibited seasonal variation, with contaminant-induced responses differing from those induced by endogenous hormones most frequently in February overall, and exhibiting additional tissue and season specificity. Exposure to the tertiary mixture induced a functional gain at the level of biochemical pathways modeling over responses induced by individual components in select tissues and seasons. Study limitations include inclusion of male goldfish only and our ability to test only small number of contaminants. It would be very relevant to examine potential sexual dimorphism in metabolic response to hormone and contaminant exposures across growth and reproductive stages by including both male and female goldfish in future studies. Overall, we demonstrate the importance of seasonally driven changes in physiology altering overall vulnerability of goldfish to metabolic perturbation induced by environmental contaminants, the relevance of which likely extends to other seasonally-breeding species.

## Data Availability

The original contributions presented in the study are included in the article/Supplementary Material, further inquiries can be directed to the corresponding author.

## Abbreviations

BPA, Bisphenol A; NP, nonylphenol; DEHP, bis(2-ethylhexyl) phthalate, fucosterol; and a tertiary mixture (DEHP + NP + FS); T, Testosterone; E2, 17β-estradiol; T3, Thyroid hormone (3,3′,5′-Triiodo-L-thyronine).
