# Supplementary figures and images for: Seasonally Related Disruption of Metabolism by Environmental Contaminants in Male Goldfish (Carassius auratus)
Source: Front Toxicol. 2021 Sep 29;3:750870. doi: 10.3389/ftox.2021.750870 (PMC8915895; doi:10.3389/ftox.2021.750870)

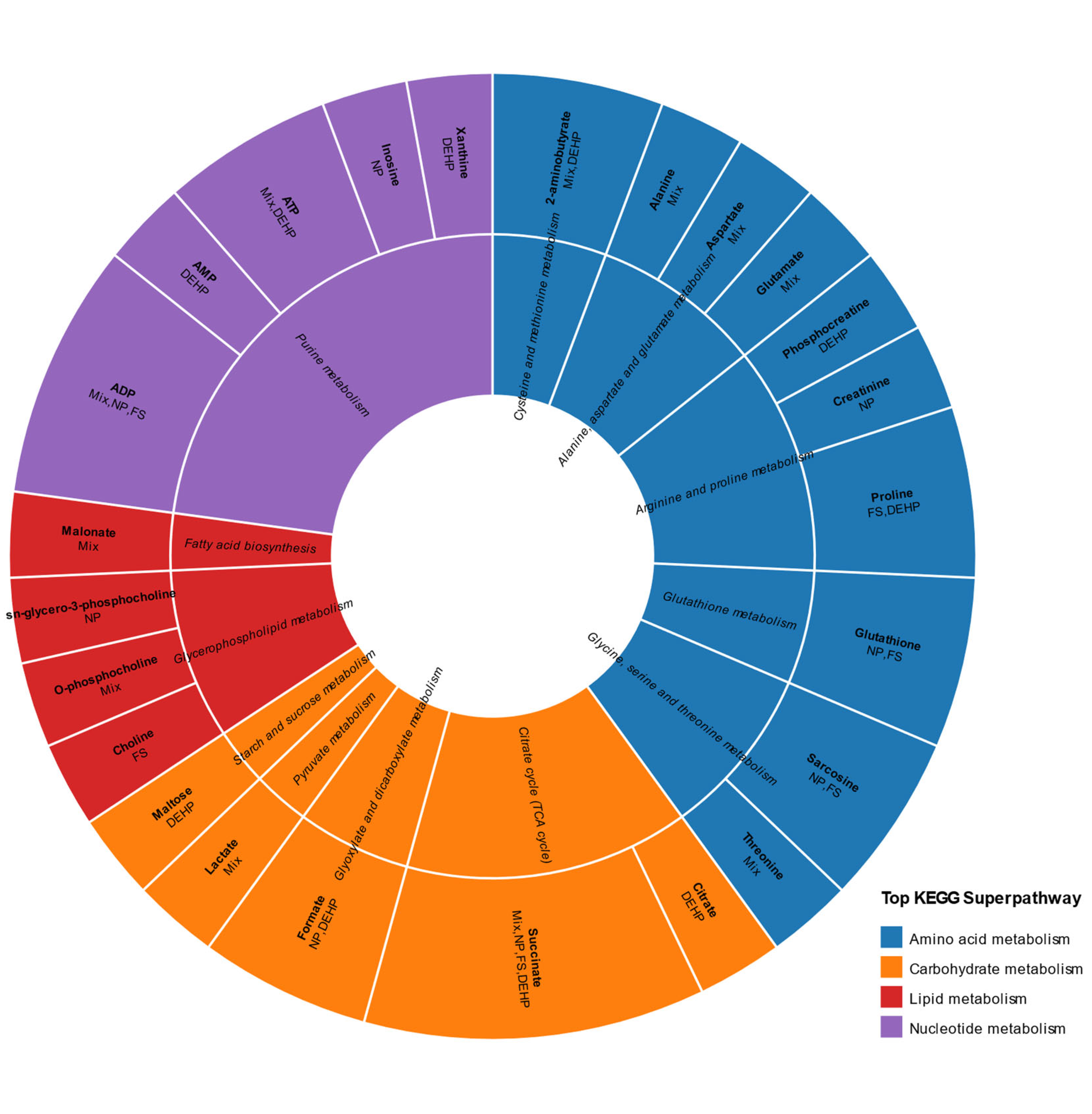

Supplement: Supplementary file 2 [file Image2.TIF]

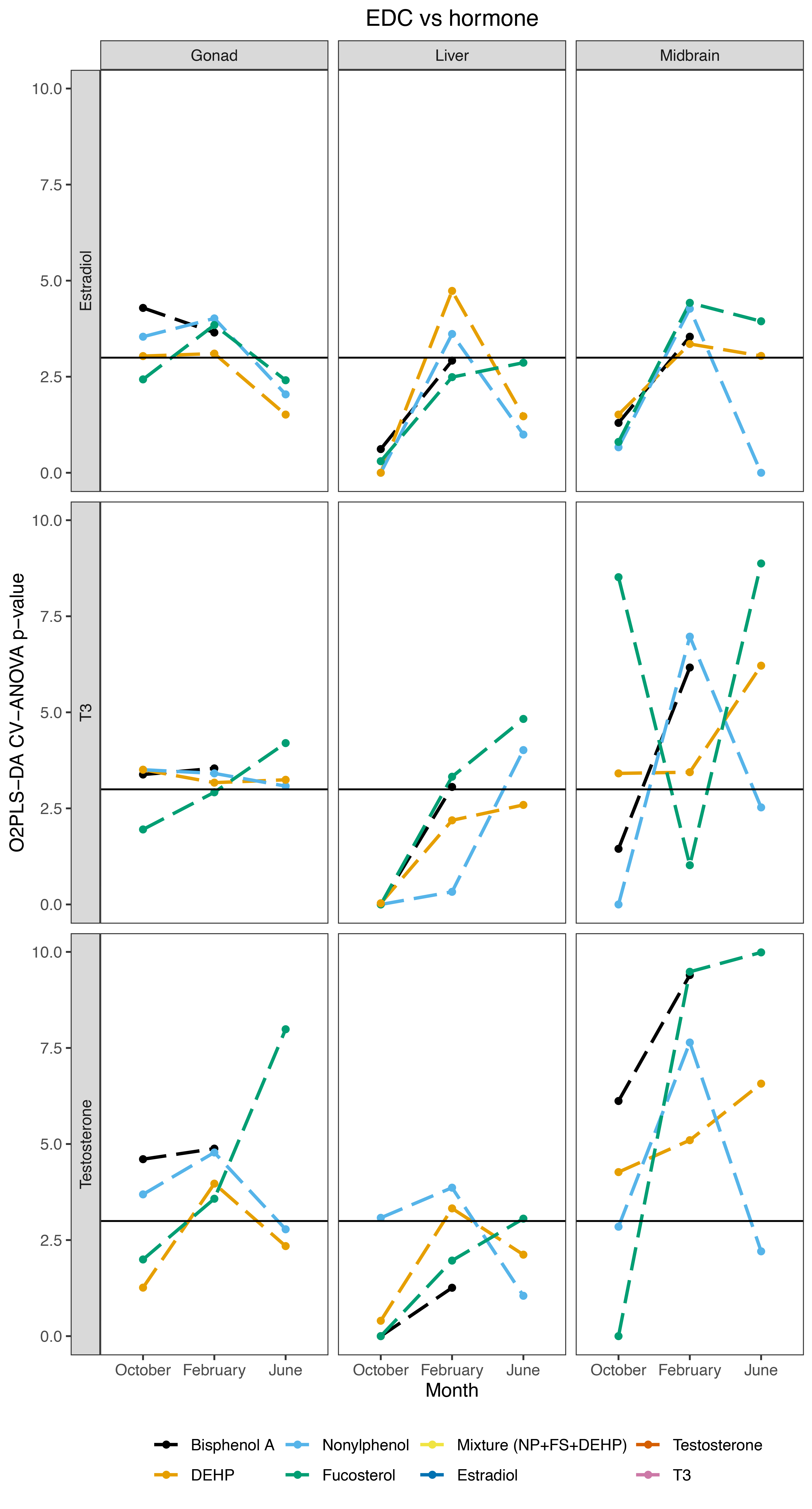

Supplement: Supplementary file 3 [file Image1.TIF]
