# Supplementary material for: Seasonally Related Disruption of Metabolism by Environmental Contaminants in Male Goldfish (Carassius auratus)
Source: Front Toxicol. 2021 Sep 29;3:750870. doi: 10.3389/ftox.2021.750870 (PMC8915895; doi:10.3389/ftox.2021.750870)
Supplement: Supplementary file 4 [file DataSheet1.docx]

Supplementary Material

# Supplementary Figures and Tables

**Contents:**

**Supplementary Table 1.** Manual KEGG annotation of altered metabolites across datasets

**Supplementary Table 2.** Relative changes in differential metabolites in midbrain, liver and gonad across seasons

Supplementary Figure 1. O2PLS-DA hormone vs. contaminant pairwise modeling summary across tissues and seasons

Supplementary Figure 2. Effect of contaminant treatments on male goldfish liver metabolome in June

Supplementary Figure 3. Treatment-specific impacts of hormone or contaminant treatments on biochemical pathways in midbrain, gonad and liver in October, February and June

**Figures additionally available as high-resolution image files.*

**Supplementary Table 1.** Manual KEGG annotation of altered metabolites across datasets

Supplementary Table 1, cont. Manual KEGG annotation of altered metabolites across datasets

Supplementary Table 2. Relative changes in differential metabolites in midbrain, liver and gonad across seasons

Supplementary Table 2, cont. Relative changes in differential metabolites in midbrain, liver and gonad across seasons

Supplementary Table 2, cont. Relative changes in differential metabolites in midbrain, liver and gonad across seasons

**
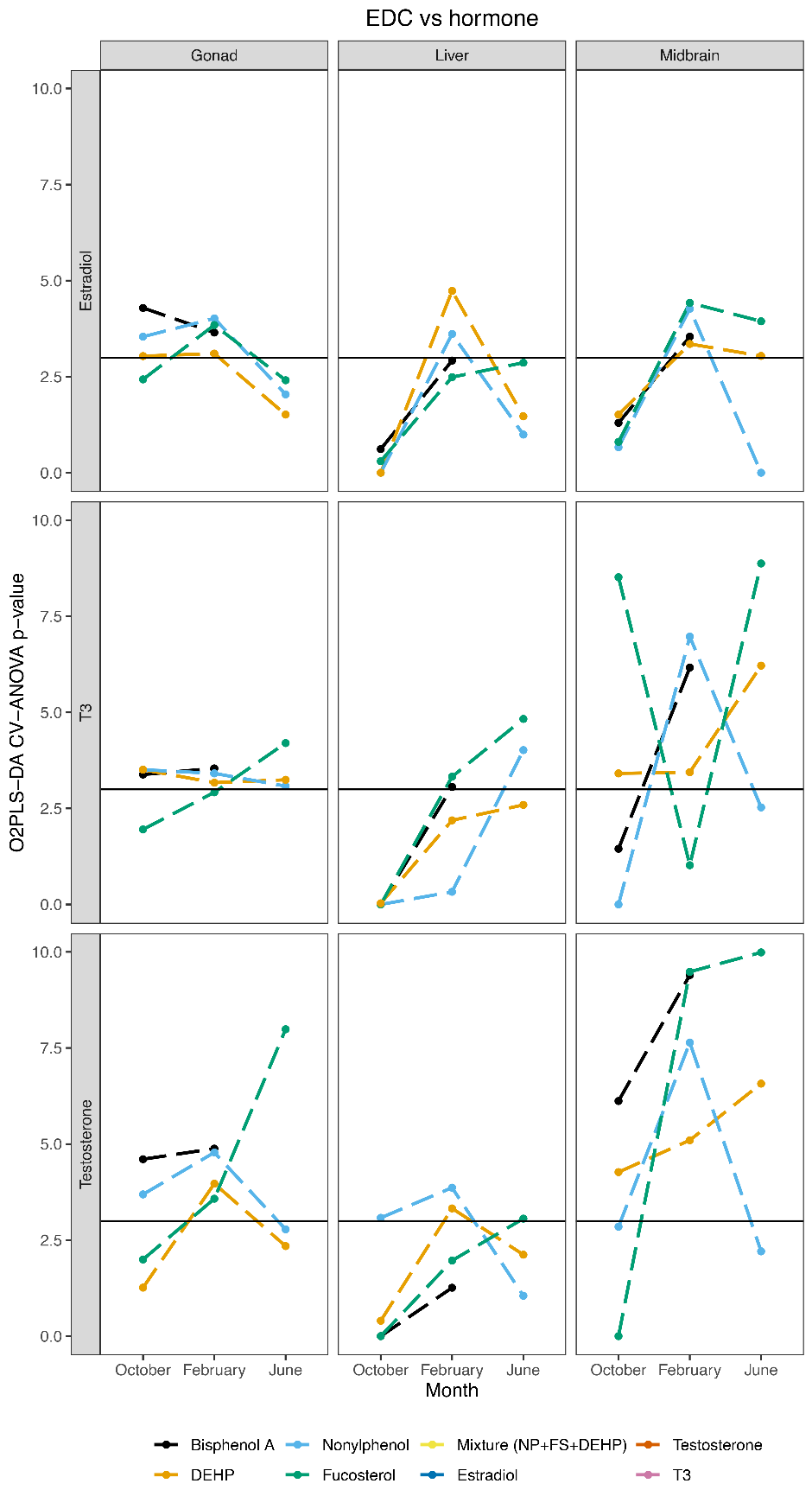
**

Supplementary Figure 1. O2PLS-DA hormone vs. contaminant pairwise modeling summary across tissues and seasons

Summary of O2PLS-DA pairwise modeling results assessing hormone vs. contaminant or mixture treatment pairs in midbrain, gonad and liver in October, February and June. CV-ANOVA *p*-values are plotted on a logarithmic scale.

**
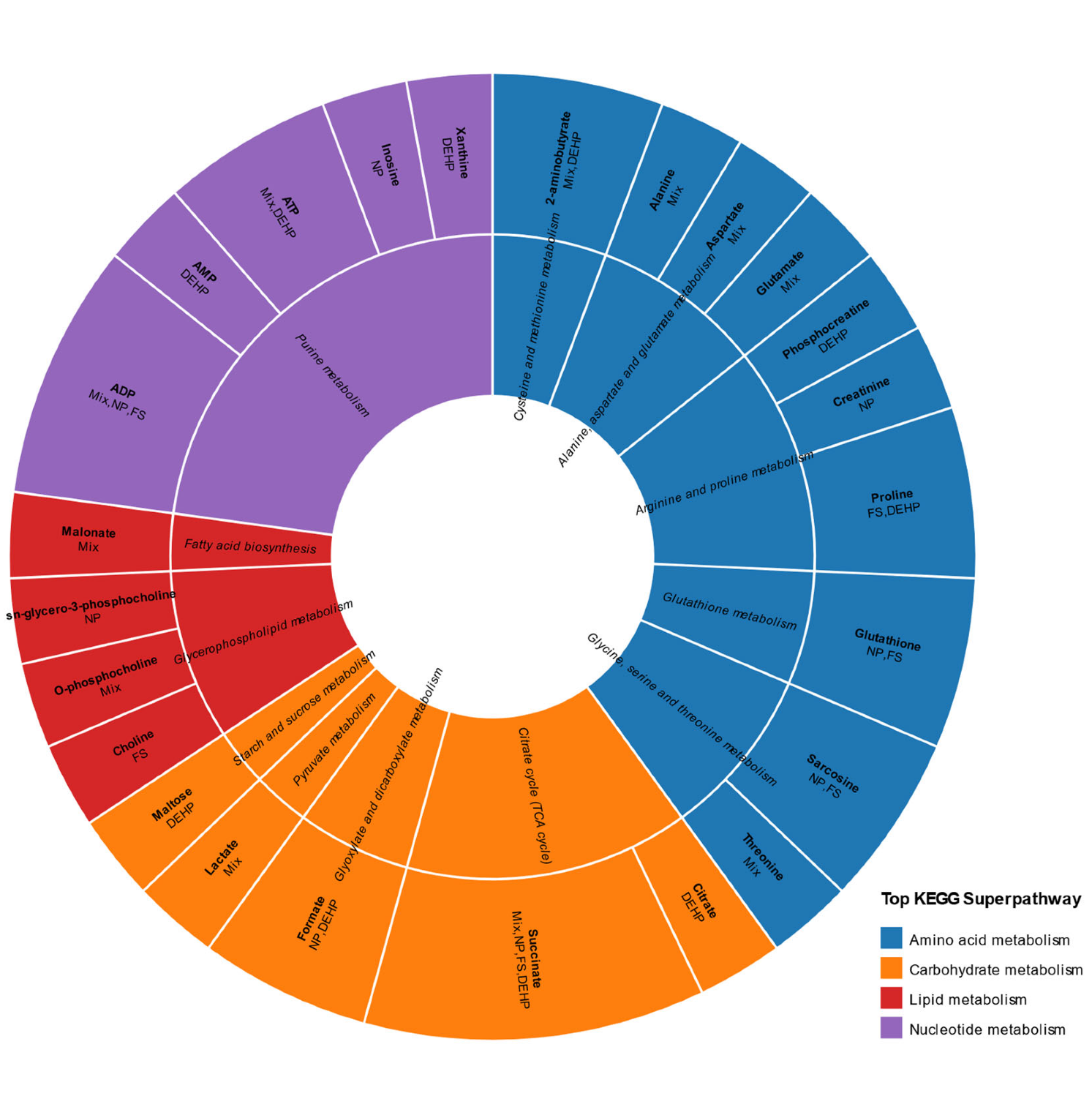
**

Supplementary Figure 2. Effect of contaminant treatments on male goldfish liver metabolome in June

Sunburst diagram depicting metabolites altered by individual contaminant and mixture exposures in liver in June and specific contaminant treatments found to alter each metabolite. Metabolites and associated biochemical pathways are color coded by KEGG superpathways.

Supplementary Figure 3. Treatment-specific impacts of hormone or contaminant treatments on biochemical pathways in midbrain, gonad and liver in October, February and June

Heatmap depicting specific biochemical pathways altered by individual hormone or contaminant exposures across tissues and seasons analyzed. Biochemical pathways are categorized by KEGG superpathways. The graded color heatmap depicts the number of treatments found to impact a biochemical pathway in a particular tissue and season, and the solid black shading indicates biochemical pathways altered by specific hormone and contaminant treatments in each tissue and season. Results are based on the Metabolite Set Enrichment Analysis (MSEA).
